# Morphologic Changes of the Intervertebral Disk During Growth

**DOI:** 10.1097/BRS.0000000000004795

**Published:** 2023-08-08

**Authors:** Aaron J.B.W.D. Moens, Joëll Magré, Moyo C. Kruyt, René M. Castelein, Steven de Reuver

**Affiliations:** a Department of Orthopedic Surgery, University Medical Center Utrecht, Utrecht, The Netherlands; b 3D Lab, Division of Surgical Specialties, University Medical Center Utrecht, Utrecht, The Netherlands

**Keywords:** intervertebral disk, annulus fibrosus, nucleus pulposus, magnetic resonance imaging, 3D segmentation, morphology, slenderness, pediatric, etiology, spinal deformities

## Abstract

**Study Design.:**

Cross-sectional.

**Objective.:**

The aim of this study was to describe morphologic changes of the annulus fibrosus (AF) and nucleus pulposus (NP) in children during growth using magnetic resonance imaging.

**Summary of Background Data.:**

Little is known of intervertebral disk (IVD) maturation as opposed to degeneration, such as changes in relative AF/NP proportions and orientation during growth. Studies suggest that IVD plays a role in the etiology of pediatric spinal deformities. Therefore, understanding the morphologic development of the AF and NP during growth is key.

**Materials and Methods.:**

An existing database of children aged 0 to 18 that had magnetic resonance imaging for indications unrelated to the spine were analyzed. The AF/NP were segmented semiautomatically from T1 to L5. The parameters: mean IVD height, cross-sectional area, slenderness (height/width ratio), volume (ratio), and relative position of the centroid of the NP within the IVD in three directions (*x*, *y*, *z*) were extracted, and compared between age, sex, and spinal level.

**Results.:**

IVD height increased modestly and predominantly in the low-thoracic and lumbar spine during the first 5 to 10 years of life. Cross-sectional area and thus volume increased steadily at all levels throughout growth. IVD slenderness decreased sharply in the first years of life and remains relatively stable throughout the remainder of growth. IVDs were smaller and more slender in females, especially in the mid-thoracic spine at early adolescence. In the upper-thoracic and mid-thoracic spine the NP comprises 10% to 12% of total IVD volume during growth, this percentage increases in the low-thoracic and lumbar spine towards 20% to 25%. In the anterior-posterior direction, the position of the nucleus increasingly shifts with age, possibly in line with the developing sagittal profile of the spine.

**Conclusion.:**

This study describes the development of thoracic and lumbar IVDs during growth and may be used as a reference for future studies on the role of IVD in the etiology of disk-related disorders.

The intervertebral disk (IVD) is a crucial component of the spine as it provides flexibility and stability.^1^ Already in 1890, John Cleland of Glasgow stated the IVD represents a modified diarthrodial or synovial joint.^2^ Until a published review of literature by Schmorl in 1928 on the herniation of the nucleus through the cartilaginous and bony endplate, the IVD was regarded as a structure of no great significance within the vertebral column.^2^ Each IVD forms a fibrocartilaginous fusion or symphysis between adjacent vertebrae and may be considered the most important spinal stabilizer. The IVD is made up of two functionally distinct regions: the outer fibrocartilaginous annulus fibrosus (AF) and the inner gel-like nucleus pulposus (NP).^3^ The AF allows limited motion between adjacent vertebrae while the NP acts as a cushion absorbing compression forces between vertebrae and providing motility to the spine. During aging, the NP becomes more and more fibrous and loses its gel-like properties.^1,4–6^

Historically, little is known of the morphologic changes that occur within the IVD during growth. Changes in the morphology of the vertebral bodies and IVD during growth have first been assessed through postmortem and radiographic studies.^7,8^ In these studies, however, IVD morphometries were approximated, since the IVD itself is not visible with radiographs. Recently, computed tomography (CT) studies also described the three-dimensional (D) morphometry of vertebral bodies during growth.^9,10^

Many spine diseases such as scoliosis, Scheuermann disease, and spondylolisthesis develop during the years that the spine, including the IVD, matures.^11,12^ Maturation involves changes that occur within the composition of the IVD cellular matrix (*e.g.* loss of water content) as well as a decrease in vascularization towards the end of trunk growth in late puberty.^13^ Part of the maturation process is a gradual ossification and ultimately fusion of the IVD to the vertebral body, thus changing the mechanical properties of the segment.^14^ During adolescence, spinal loading tremendously increases over a short period of time due to rapidly increasing body mass and moment arm. For harmonious development, bone, and disk maturation must be in synchrony with that rapidly increasing spinal loading. How the IVD adapts to this rapid change in loading and its individual variations is unknown but of specific interest in understanding the initiation of spinal deformities during growth. Therefore, the main aim of this study is to accurately describe morphometric changes within the IVD and the relative volumetric contribution during growth in an asymptomatic population, based on spinal magnetic resonance imaging (MRI).

## MATERIALS AND METHODS

### Study Population

In this cross-sectional study, an existing database of T2-weighted sagittal MRI scans of pediatric patients (aged 0–18 yr) was used. Due to anonymization, the precise scan indication is unknown. MRI scans dated from October 2011 to January 2019 and were made in a single tertiary children’s hospital with a 1.5 T Philips Achieva MRI scanner (slice thickness 3–4 mm). Exclusion criteria were: scans indicating any spinal pathology, thoracic and lumbar spine not fully imaged, inability to distinguish the AF from the NP properly (*e.g.* due to poor scan quality or slice thickness >4 mm), malposition of the patient (*i.e.* hyperextended or rotated), no spinal MRI (*e.g.* brain MRI) and if a sagittal series were not available (Table 1).

**TABLE 1:** Excluded Scans

Exclusion criterium	Excluded scans (N=54)
Spine not fully imaged	13
No sagittal MRI available	6
Poor scan quality	11
Spinal pathology	16
Malposition	1
Brain MRI	3
Improper slice thickness	4

MRI indicates magnetic resonance imaging.

### Segmentation Protocol

**Figure 1:**
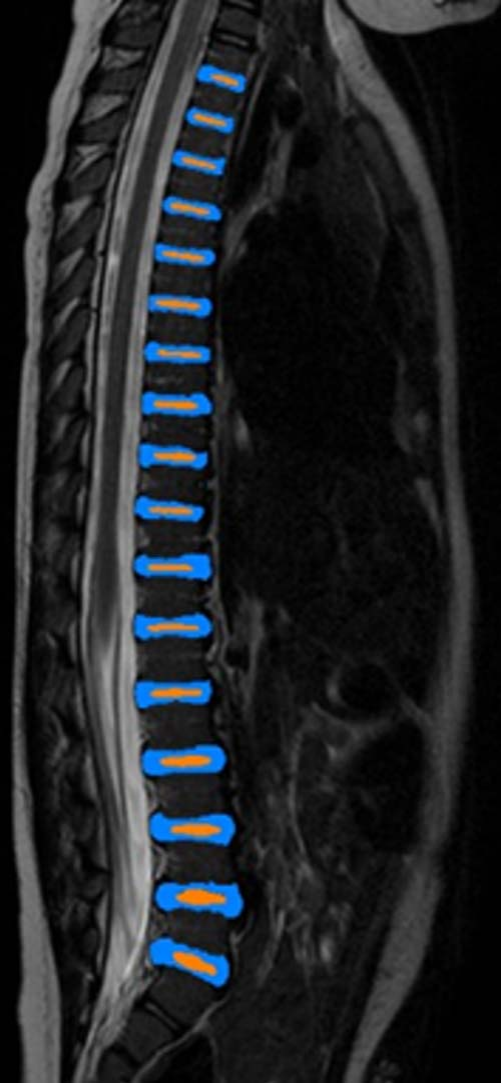
T2-weighted magnetic resonance imaging scan. Example of a segmentation of T1–L5. Nucleus pulposus in orange and annulus fibrosus in blue.

One trained observer segmented the AF/NP of the spinal levels T1–L5 (Figure 1) using Mimics software (v24.0; Materialise).^15^ The NP was identified as a relatively hyperintense (water-rich) signal on T2-weighted images. First, the NP was segmented semiautomatically using thresholding to create a mask, highlighting the NP of each IVD. Since the gray values differed individually, the mask thresholds were determined by the observer specifically for each MRI scan to maximize NP coverage. A second mask was created by setting a new threshold to invert the previous mask of the NP, highlighting both the AF and surrounding tissues. The AF was then segmented through interpolated thresholding and visually identifying the AF and highlighting its contour manually. This was performed for each AF between T1 and L5 in every sagittal slice. Finally, the segmentations were exported to 3-Matic (v15.0; Materialise) and refined using the “wrap” tool, which results in smoothing out the model and filling in minor gaps (<1 mm) in the segmentation.

### Data Extraction

**Figure 2:**
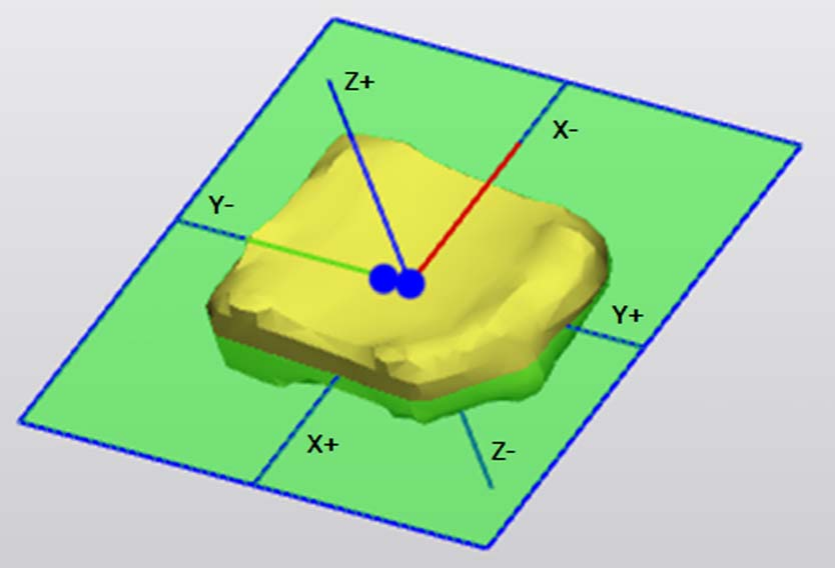
Nucleus orientation analysis. A new coordinate system (*x*, *y*, *z*) was plotted in the center of both the intervertebral disk and nucleus pulposus. The relative center of the nucleus pulposus compared with the center of the total intervertebral disk was plotted and the distance between them calculated in three directions.

In 3-Matic, the volumes (mm^3^) of the AF and NP were extracted from the 3D models.^16^ The total volume of the IVD (V_IVD_) was calculated by adding up the volumes of the NP (V_NP_) and AF (V_AF_). The ratio between V_NP_ and V_IVD_ (volume ratio) was calculated. Measurements were stratified into four spinal levels: upper-thoracic (T1–T4), mid-thoracic (T5–T8), lower-thoracic (T9–T12), and lumbar (L1–L5) according to SRS definitions and measurement manual.^17^ Furthermore, a custom variable as a proxy for slenderness of an IVD, was calculated as the height divided by the square root of the cross-sectional area. To correct for rotation as a consequence of patient orientation in the MRI scanner a new sagittal plane was defined by creating a plane through the centers of gravity of the AF of T1, L1, and the most posteriorly positioned AF of the patient, regardless of its sagittal profile. A separate coordinate system was defined for each AF individually by their 3 inertia axes; *x*-axis (left-right), *y*-axis (anterior-posterior), and *z*-axis (cranial-caudal). These individual coordinate systems were rotated around their *z*-axis until the *y*-axis was in line with the sagittal plane (Figure 2). IVD height in the center of gravity (mm), and cross-sectional area (mm²) through the true transverse plane (xy-plane) of the individual IVD were extracted from 3-Matic. The distance between the center of gravity of the NP and IVD as a whole in the three inertia axes was calculated for each IVD. Measurements were combined in three age groups (0–3/infantile, 4–10/juvenile, and 11–18/adolescent), as defined by the Scoliosis Research Society.^18^

### Statistical Analysis

Simple scatterplots were made for V_AF_, V_NP_, volume ratio (V_NP_:V_IVD_), mean IVD height, cross-sectional area, and slenderness *versus* age for males and females. To visualize the change in IVD morphologic parameters during growth, a locally estimated scatterplot smoothing curve was fitted to each plot. Simple logistic regression was performed to test changes in IVD morphology with age. Changes in IVD morphology with increasing age were assessed with simple logistic regression, while differences between males and females were assessed with a multivariate regression analysis. A post hoc *t* test was done for disk slenderness of the mid-thoracic spine between boys and girls aged 9 to 13, given the relevance of this potential difference for the etiology of scoliosis. Intraoperator and interoperator variability were assessed in 3 MRIs (102 individual segmentations) for the segmentation of the AF and NP separately and for the IVD as a whole. Values >0.75 indicate good reliability and values over 0.90 excellent reliability.^19^ Statistical significance was set at 0.05. All statistical analyses were performed with SPSS 27.0 for Windows (IBM Corp.).^20^

## RESULTS

### General

From 180 eligible MRI scans in the database, a total of 126 were included. A total of 4284 segmentations were included and analyzed (*i.e.* both the AF and NF of 2142 IVDs). Intraoperator and interoperator variability for segmentation of the AF, NF, and IVD, intraclass correlation coefficients were >0.94 (Table 2).

**TABLE 2:** Interobserver and Intraobserver Variability*

	IVD	AF	NP
Interobserver ICC	0.98 (0.98–0.99)	0.98 (0.93–0.99)	0.96 (0.91–0.98)
Intraobserver ICC	0.96 (0.94–0.97)	0.94 (0.89–0.97)	0.98 (0.95–0.99)

* Values are expressed as ICC (95% CI).

AF indicates annulus fibrosus; ICC, intraclass correlation coefficient; IVD, intervertebral disk; NP, nucleus pulposus.

### IVD Height and Cross-sectional Area

**Figure 3:**
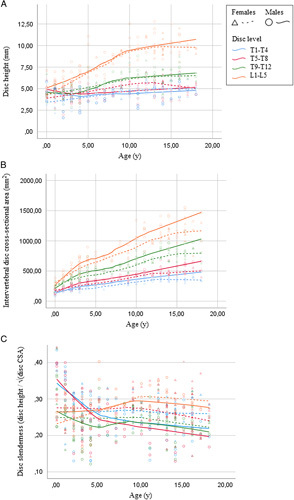
Scatterplot of mean intervertebral disk height (mm) (A), mean intervertebral disk cross-sectional area (mm²) (B), and intervertebral disk slenderness (C) of four intervertebral intervertebral disk level groups by age in males and females. Each dot represents the mean value at a intervertebral disk level in a single person. A Loess regression line is fitted for each intervertebral disk level. CSA indicates cross-sectional area.

Disk height predominantly increased in the low-thoracic and lumbar spine during the first 5 to 10 years of life, with males having a marginally higher mean IVD height than females. The IVD height in the upper-thoracic and mid-thoracic spine increased slightly to 5 mm in early adulthood. In the low-thoracic spine, height increased from 4 to 7 mm, and in the lumbar spine from 5 to 10 mm. After the age of 10 IVD height remains relatively stable. The IVD cross-sectional area showed a consistent increase throughout growth at all spinal levels and was overall significantly greater in males than females. The increase in cross-sectional area from birth to early adulthood was greater in males *versus* females (460% *vs.* 325% for all spinal levels combined). Cross-sectional area growth increased least from birth to early adulthood in the upper-thoracic spine (males 420% *vs.* females 250%) and most in the lumbar spine (males 520% *vs.* females 390%). Absolute values per age and spinal level are presented in Figure 3A and B.

### Disk Slenderness

In general, the slenderness of the IVD tended to decrease sharply during the first years of life, increased slightly at ages 5 to 9, and decreased again steadily for the remainder of growth (Figure 3C). Overall, females had more slender IVDs compared with males (*P*=0.025). This was most prominent for the mid-thoracic area of children aged 9 to 13 where the IVDs of females were remarkably more slender (0.23 *vs.* 0.27, *P*<0.001).

### IVD Volume

**Figure 4:**
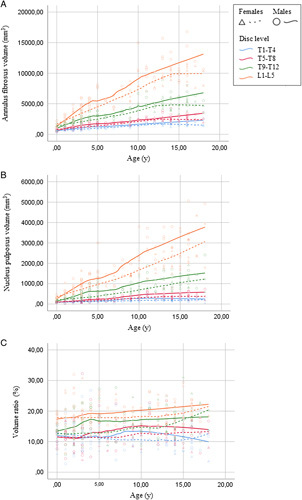
Scatterplot of mean annulus fibrosus volume (mm^3^) (A), mean nucleus pulposus volume (B), and ratio of nucleus to intervertebral disk volume (%) (C) of four intervertebral intervertebral disk level groups by age in males and females. Each dot represents the mean value at a intervertebral disk level in a single person. A Loess regression line is fitted for each intervertebral disk level.

The IVD volume increased consistently throughout growth. Males showed a significantly higher V_AF_ and V_NP_ than girls, at all IVD levels, at any age. V_AF_ and V_NP_ both increased more caudally and more in males than in females. From birth to early adulthood, V_AF_ increased in the upper-thoracic area with 380% in males and with 260% in females, whereas in the lumbar spine, this was 980% and 700%, respectively. While absolute NP volume is smaller than AF volume, the relative volume increase of the NP was greater. From birth to early adulthood, V_NP_ increased in the upper-thoracic region with 360% in males and with 300% in females, whereas in the lumbar spine, this was 1320% and 1180%, respectively. Absolute values per age and spinal level are presented in Figure 4A and B.

The volume ratio (V_NP_:V_IVD_) in the upper-thoracic and mid-thoracic spine remained relatively stable throughout growth in both males and females, with the V_NP_ comprising around 10% to 12% of the V_IVD_. The volume ratio increased slightly with age, mostly in the low-thoracic and lumbar spine in both males and females. For males, this increase was low-thoracic from 14% to 19%, and lumbar from 18% to 23%. For females, similar values were observed, as the volume ratio increased low-thoracic from 12% to 20% and lumbar from 17% to 25%. Absolute values per age and spinal level are presented in Figure 4C.

### Nucleus Position

**Figure 5:**
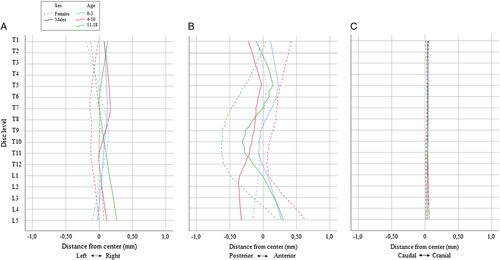
Relative change in center of mass of the nucleus pulposus within the intervertebral intervertebral disk throughout the spine, in three age groups, for males and females. Fitted with Loess regression lines, (A), change in the left-right orientation; (B), change in the anteroposterior orientation; (C), change in the craniocaudal orientation.

There was very little nucleus offset in left/right orientation at all ages and spinal levels, and there was no nucleus deviation at all in the caudal/cranial direction. However, deviation of the nucleus was most apparent in the anterior/posterior direction, with differences among males and females and between age groups. In infantiles, this male-female difference was less pronounced and the nucleus fairly centered, but in juveniles and more so in adolescents, the nucleus was positioned towards the convexity of the sagittal curve, anteriorly in the lumbar spine, posteriorly at the mid-thoracic levels, and back to neutral in the upper-thoracic spine. The absolute values for the changes in the NP centroid can be seen in Figure 5.

## DISCUSSION

Semiautomatic 3D segmentation of MRI scans was used to cross-sectionally describe morphologic changes of and within the IVD during growth in children from 0 to 18 without manifest spinal disorders.

IVD height increases mainly in the low-thoracic and lumbar disks and predominantly occurs in the first 5 to 10 years of life. The cross-sectional area of the IVD and thus the volume increased consistently during growth. IVD slenderness, as a proxy for mechanical stability, decreases most sharply in the first years of life, then tends to increase slightly and decrease again steadily after the age of ∼10 years. This is due to disk height increasing solely in the first 10 years of life and then remains stable, whereas cross-sectional area increase is sharpest in the first 3 to 4 years of life and then increases at a slightly slower pace. In a recent biplanar radiographic study by Vergari *et al* ^21^ and a CT analysis by Chen *et al*,^22^ it was suggested that IVD slenderness may be a risk factor for scoliosis. Idiopathic scoliosis manifests itself mostly in females and usually has its onset in early adolescence.^23^ Furthermore, it has been demonstrated that the spine in patients with scoliosis is more slender compared with the healthy population.^21,22^ An interesting finding in the current study was that female IVDs in the mid-thoracic spine at early adolescence were significantly higher and narrower, thus more slender than those of males, as well as when compared with other areas of the female spine. This may indicate that females, especially in the mid-thoracic spine during adolescence, have a phase of relatively decreased mechanical stability assuming all other variables are evenly distributed. Subsequently, this could play a role in the overrepresentation of females in adolescent idiopathic scoliosis, mostly developing mid-thoracic curves.^23^ However, this suggestion based on cross-sectional descriptive data remains uncertain and requires a prospective longitudinal confirmation or rejection.

During growth we observed that the nucleus orientation is very centered in the right-left and cranial-caudal direction, however in the anterior-posterior direction, the nucleus increasingly shifts with age, following the known development of the spinal sagittal profile, developing from a slight kyphosis in infants towards a more defined S-shape in adolescents^24^ of the spine. This indicates that the NP dynamically adapts to the shape of the disk and resultant pressure distribution. While this effect was not statistically significant, most likely because the deviation is very slight and this descriptive study was not powered for this outcome, these results may indicate the NP is subjected to the deformation of the IVD as part of the developing kyphosis and lordosis of the spine. However, a future study should confirm or reject this hypothesis.

The current study confirms the earlier CT-based findings that spinal height growth is mainly in the vertebral bodies and much less in the disks. Novel data from this study demonstrate that growth patterns of the NP and AF appears to be similar in boys and girls, although V_AF_ seems to plateau at an earlier age in girls, probably due to the fact that girls enter their growth spurt at an earlier age. There were no distinct growth patterns of the absolute or relative V_AF_ or V_NP_ in boys and girls at their expected age of peak height velocity, which is around 13 to 14 for boys and 11 to 12 for girls.^25^ This absence of differences could be due to the limited number of included participants at certain ages, or the fact that the skeletal maturity and age from maturity offset of included participants were unknown.

The nucleus comprises 10% to 12% of the IVD volume in the upper-thoracic and mid-thoracic spine and remains relatively stable throughout growth. In both males and females, this ratio increased to around 20% in the low-thoracic and 25% in the lumbar spine in early adulthood. In other studies, the ratio between V_NP_ and V_IVD_ or V_NP_ and V_AF_ is used as an indicator for IVD hydration.^13,26^ Bolzinger *et al* ^13^ hypothesized that lower IVD hydration in girls might be a contributing factor in the etiology of idiopathic scoliosis and studied the hydration status of nonpathologic lumbar IVDs in a pediatric population. They reported a nonsignificant increase of IVD hydration by age and no statistical influence of sex. In this study, disk hydration tended to be slightly lower in females compared with males.

We recognize certain limitations of the current study. The indication for the MRI scan was unknown due to anonymization of patient data as required by the privacy legislations. While most spinal pathology could easily be detected, specific patient conditions or diseases influencing the spine cannot be ruled out. Second, the segmentation becomes more difficult with smaller volumes and thus may bias the thoracic IVD measurements. Third, segmentation of the lateral IVD borders was limited by the slice thickness of the MRI scan, which was 3 to 4 mm in all cases. While the effects of this are random, it could result in an underestimation of IVD transverse cross-sectional surface area, and thus volume. Furthermore, it may also have a minor effect on the calculation of the change in nucleus position within the IVD in the left-right direction throughout growth. Finally, for some age groups, only a limited number of scans were available (n=3 for ages 6, 8, 17, and 18) This may distort the mean value and also impact the significance of results.

## CONCLUSIONS

IVD height increases minimally during growth in the low-thoracic and lumbar spine, whereas the transverse cross-sectional area and thus volume increases consistently at all spinal levels. IVD slenderness decreases sharply in the first years and more steadily thereafter. Female IVDs, especially mid-thoracic and in early adolescence, were significantly more slender than males. In the upper-thoracic and mid-thoracic spine, the NP comprises 10% to 12% of total IVD volume. This increases in the low-thoracic and lumbar area to 20% to 25% at the beginning of adulthood. Anterior-posteriorly, the NP shifts slightly following the sagittal profile, becoming more pronounced with age.

Key Points

- IVD height moderately increased in the low-thoracic and lumbar spine during the first 5 to 10 years of life, while the IVD height remained relatively stable in the upper-thoracic and mid-thoracic spine.
- IVD transverse cross-sectional area and volume increase consistently. IVD volume was significantly larger in males at all levels and ages. The ratio of NP volume to IVD volume increases predominantly in the low-thoracic and lumbar spine in both males and females towards 20% to 25% at the beginning of adulthood, while remaining stable in the upper-thoracic and mid-thoracic region at 10% to 12%.
- IVD slenderness decreases sharply in the first years of life and remains relatively stable afterwards. Female IVDs, especially mid-thoracic and in early adolescence, were more slender than those of males.
- This MRI-based morphometric map describing the normal development of thoracic and lumbar intervertebral IVDs during growth may be used as a reference in future studies to help understand the role of IVD in the etiology of disk-related disorders.
